# Genetic manipulation for the non-model protozoan *Eimeria*: Advancements, challenges, and future perspective

**DOI:** 10.1016/j.isci.2025.112060

**Published:** 2025-02-17

**Authors:** Yaru Li, Jingxia Suo, Ruiying Liang, Lin Liang, Xianyong Liu, Jiabo Ding, Xun Suo, Xinming Tang

**Affiliations:** 1 Key Laboratory of Animal Biosafety Risk Prevention and Control (North) & Key Laboratory of Veterinary Biological Products and Chemical Drugs of MARA, Institute of Animal Science, Chinese Academy of Agricultural Sciences, Beijing 100193, China; 2 National Key Laboratory of Veterinary Public Health Security, Key Laboratory of Animal Epidemiology of the MARA, National Animal Protozoa Laboratory & College of Veterinary Medicine, China Agricultural University, Beijing 100193, China

**Keywords:** Techniques in genetics, Parasitology

## Abstract

*Eimeria* parasites pose a significant global threat to animal health, necessitating improved and cost-effective control measures. Genetic manipulation is pivotal for understanding *Eimeria* biology and designing targeted control strategies. Recent advancements, including genome sequencing and the development of transient and stable transfection systems, have significantly enhanced insights into the molecular biology of *Eimeria*. These advancements have paved the way for cutting-edge techniques like CRISPR-Cas9 gene editing. This review summarizes the key milestones in the development of genetic manipulation platforms for *Eimeria* and their transformative applications, such as the development of next-generation drugs, vaccines, and *Eimeria*-based vaccine vectors. Furthermore, this review provides insights that could be applicable to the establishment of genetic tools for other protozoan organisms.

## Graphical abstract

**Figure:**
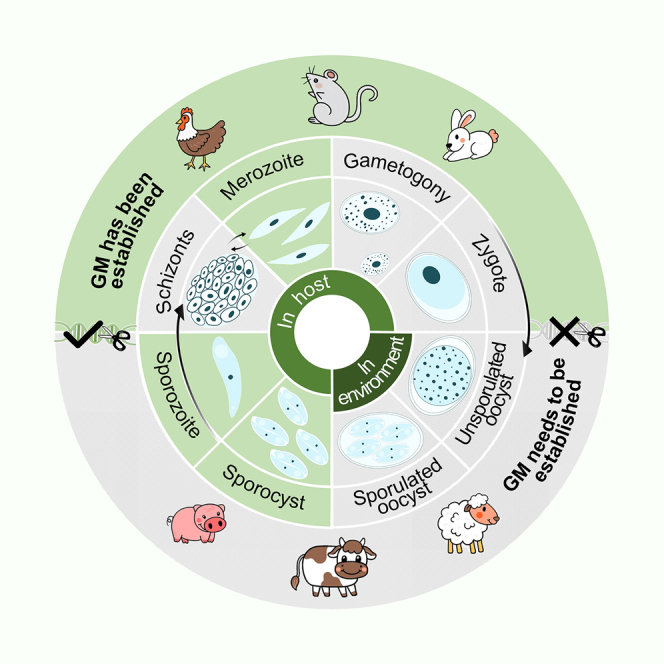

## Coccidiosis: A globally distributed animal parasitic disease needs novel control methods

Coccidiosis is one of the most prevalent parasitic diseases in animals, mainly caused by protozoan parasites belonging to the genus *Eimeria*.^1^ The primary symptom is diarrhea, which can become bloody in severe cases. Both clinical and subclinical forms of coccidiosis, classified based on the severity of clinical symptoms, significantly impact the health, productivity, and economic outcomes of livestock.^1^^,^^2^ According to recent estimates, chicken coccidiosis alone results in an annual economic loss of approximately $10 billion globally.^3^ Although there is no comprehensive estimation system for other poultry (such as turkeys, ducks, and pigeons) or for rabbits, sheep, and cattle, the economic losses due to coccidiosis in these industries are likely substantial.^4^ Therefore, effective coccidiosis control measures are essential, particularly in modern animal husbandry, to minimize economic losses and ensure healthy livestock production.

Anticoccidial drugs are currently one of the primary methods for controlling coccidiosis.^5^^,^^6^ However, the drawbacks associated with the routine use of these chemoprophylactic agents have become increasingly apparent: (1) Widespread drug resistance: Intensive and prolonged use of anticoccidial drugs has led to the emergence of drug-resistant strains, rendering the drugs less effective over time.^7^ Alarmingly, resistance to some anticoccidial drugs has been reported within just a year of their introduction, and resistance has been documented for all anticoccidial drugs currently available on the market.^8^ As a result, the effective use or strategic planning of anticoccidial drugs is becoming increasingly limited. (2) Food safety concerns: The public’s growing concerns over food safety, particularly related to drug residues in animal products, have also led to restrictions on the use of anticoccidial drugs.^9^^,^^10^ This concern limits their application in food production, as consumers demand safer, residue-free food products. (3) Environmental impact: Anticoccidial drugs contribute significantly to environmental drug residues, which is a growing environmental concern.^11^^,^^12^ Consequently, governments and regulatory bodies are increasingly advocating for the reduction in the use of these drugs to mitigate their environmental impact. These challenges underscore the need for alternative and more sustainable approaches to coccidiosis control in modern animal husbandry.

Vaccination with live parasites, coupled with proper husbandry practices, is another effective strategy for controlling coccidiosis.^2^ Currently, only coccidiosis vaccines for chickens and turkeys have been commercialized globally,^2^^,^^13^ leaving a significant gap in the availability of vaccines for other animals such as rabbits, pigeons, sheep, and cattle. The production of existing live attenuated or non-attenuated vaccines is constrained by their reliance on live animals, which limits their production capacity. Additionally, one of the drawbacks of live vaccine immunization is the potential impact on the performance of poultry.^14^ This is particularly true for vaccines containing virulent strains, which carry the risk of causing coccidiosis.^2^ To address these challenges, there is an urgent need for new vaccine concepts. These include the knockout of virulence genes to enhance vaccine safety and the use of genetic manipulation to improve the immunogenicity of vaccine components. Such innovations are essential to meet the demands of intensive and sustainable breeding industries while effectively preventing and controlling coccidiosis.

## Biological characteristics of *Eimeria*: The foundation for genetic manipulation

*Eimeria* is a genus of parasitic protozoa belonging to the phylum Apicomplexa.^1^ These parasites are known for causing coccidiosis, a significant disease in poultry and other livestock. Over 1900 species of *Eimeria* have been reported in animals. *Eimeria* species have a complex life cycle that includes both sexual and asexual reproduction, occurring in distinct phases within the host and the external environment. *Eimeria* species exhibit host specificity, with different species infecting particular hosts and even specific tissues within the host.^1^ Understanding the biology of *Eimeria* is crucial for developing effective control measures, including vaccines and other therapeutic strategies, especially the establishment of the genetic manipulation platform.

### Life cycle

**Figure 1:**
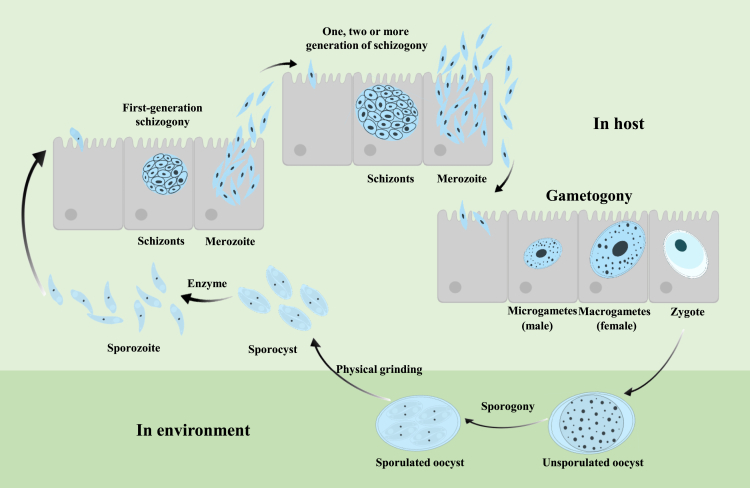
Overview diagram of the *Eimeria* life cycle The life cycle of *Eimeria* involves stages both in the host and in the environment. In the host, the parasite undergoes multiple rounds of schizogony, starting with the first-generation schizonts, which release merozoites. Merozoites invade host epithelial cells and proceed through one or more additional generations of schizogony. Following this, gametogony occurs, producing male microgametes and female macrogametes, which fuse to form a zygote. The zygote is excreted into the environment as an unsporulated oocyst. In the environment, the oocyst undergoes sporogony to form a sporulated oocyst, containing infectious sporozoites within sporocysts. Sporozoites are released from oocysts through physical grinding and chemical digestion, enter intestinal epithelial cells, and begin the next generation life cycle.

The life cycle of *Eimeria* includes both asexual and sexual reproduction stages and occurs within the host and the external environment (Figure 1). The life cycle begins when unsporulated oocysts are excreted in the feces of the infected host.^1^ These oocysts undergo sporulation in the environment, given the suitable conditions (adequate oxygen, temperature, and humidity). Sporulated oocysts contain infective sporozoites. Therefore, unsporulated oocysts are the preferred recipient cells for genetic manipulation, as the corresponding phenotype can be obtained after a short period of *in vitro* culture. However, the structure and composition of the oocyst wall of *Eimeria* differs significantly from the cell membrane of mammalian cells and the cell wall of plant cells, making it difficult to introduce foreign genetic materials.^15^ To date, an effective transfection system for *Eimeria* oocyst has not been established.

When sporulated oocysts are ingested by a susceptible host, sporozoites are released through a combination of physical disruption and the chemical action of bile and digestive enzymes, enabling them to invade intestinal epithelial cells. *In vitro*, sporulated oocysts can be mechanically disrupted by grinding or shaking to release sporocysts, which are then treated with bile and trypsin, or other digestive enzymes to yield sporozoites with cell-invasion capabilities.^16^ Sporozoites are the preferred recipient cells for genetic manipulation and *in vitro* research of *Eimeria*.^17^^,^^18^ The sporozoites invade the epithelial cells of the intestine, where they undergo multiple rounds of asexual reproduction, producing schizonts that release large quantities of merozoites.^19^ Merozoites can also be easily separated and purified from host cells while maintaining their ability to reinvade host cells.^20^ For this reason, they are also used as recipient cells for genetic manipulation.^21^^,^^22^ The merozoites then differentiate into sexual stages—microgametocytes/microgametes (male) and macrogametocytes/macrogametes (female). Fertilization results in the formation of zygotes, which develop into new oocysts. Oocysts are shed from host cells and are then excreted with feces (Figure 1).

### Bioinformatics

Bioinformatics is an interdisciplinary field that involves the systematic collection, processing, storage, dissemination, analysis, and interpretation of biological data. By integrating principles from biology, computer science, and information technology, it aims to decipher the complex biological information embedded in extensive datasets.^23^ Recent studies have revealed important insights into the genome of *Eimeria* species, especially in relation to their size, structure, and complexity. As *Eimeria* are apicomplexan protozoa, their genomes share some common features with other parasites in the group, but also exhibit unique traits. *Eimeria* genomes are relatively compact compared to other apicomplexans, with sizes ranging from ∼8 to 13 Mbp, depending on the species. The genome is organized into multiple chromosomes (usually 7–9), each carrying clusters of genes involved in processes like protein synthesis, immune evasion, and cellular invasion.^24^ Comparative genomic analyses have shown that while *Eimeria* shares many features with other apicomplexans like *Plasmodium* and *Toxoplasma*, it also exhibits distinct genomic adaptations. These include the expansion of gene families involved in host cell invasion and the immune response. Promoter regions in *Eimeria* are typically short (∼200–500 bp) and are tightly linked to gene expression, enabling the parasite to switch between different functional phases.^24^^,^^25^ The reference genome of *Eimeria tenella* has paved the way for a deeper understanding of the genetic mechanisms governing key traits. For example, resequencing technologies have pinpointed mutation sites in resistance genes of drug-resistant strains.^6^^,^^26^ Additionally, comparative transcriptomics has been employed to identify regulatory genes involved in *Eimeria* growth and development stages, such as schizogony and gametogony, with the aim of uncovering the genetic basis of *Eimeria* precocity.^27^^,^^28^^,^^29^ It is crucial to note that while existing databases have published raw genomic data for other chicken coccidia species, further refinement and enhancement of these genomes remain essential.

## Advancements in genetic manipulation: transfection, selection, and evaluation systems

Genetic manipulation is a fundamental tool for advancing biological research. As previously discussed, the establishment of a genetic manipulation platform for *Eimeria* requires a multidisciplinary and multi-technological approach. However, the progress in developing such platforms has been relatively slow due to limitations like the *in vitro* culture systems and the quality of the *Eimeria* genome.

### Transfection construct design

**Figure 2:**
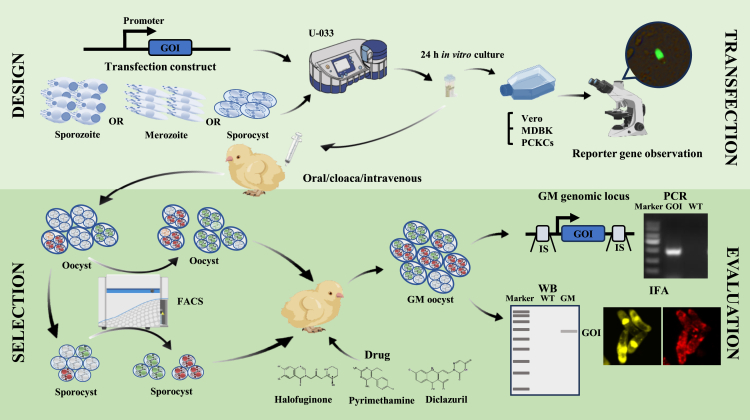
Schematic for the development of genetically modified (GM) *Eimeria* oocysts The process of gene editing in *Eimeria* species and the subsequent evaluation of GM oocysts. **Transfection and cultivation:** The gene of interest (GOI), driven by a specific promoter (as detailed in Table 1), is delivered into the *Eimeria* genome via electroporation using a shuttle plasmid. This process involves the use of sporozoites, merozoites, or sporocysts as recipient cells, and may employ systems like the U-033 electroporation system. After the electroporation, the GM parasites are cultured *in vitro* for 24 h or more using cell lines such as Vero, MDBK, or PCKCs. The presence and expression of the reporter gene are then observed under a microscope. ***In vivo* application**: The transfected sporozoites, merozoites, or sporocysts are administered to chickens via oral, cloacal, or intravenous routes depending on the species and parasitic site of *Eimeria*. When drug resistance genes are present, the corresponding drug should be included in the first-generation application. It’s crucial to note that to ensure the expression of drug resistance genes in the transfected parasites, the drug is typically added 18 to 24 h after inoculation. **Selection and propagation**: After obtaining genetically modified oocysts, it is essential to perform reporter gene detection. Depending on the type of fluorescent reporter gene used, select the appropriate excitation wavelength for the flow cytometry channel to sort the oocysts or sporocysts. These genetically modified oocysts or sporocysts can then be enriched and subjected to further passage and amplification. When utilizing drug resistance genes, the addition of the corresponding drug can significantly enhance selection efficiency. It has been demonstrated that drug resistance genes for pyrimethamine, halofuginone, and diclazuril (unpublished data) are effective in screening GM *Eimeria* oocysts. **Evaluation of GM oocysts**: After stabilizing a population of GM oocysts, various identification methods can be employed to verify the modifications. At the genetic level, PCR can confirm the stable presence of the gene of interest, while techniques such as genome walking or plasmid rescue can analyze the genomic insertion site. Due to potential challenges in RNA extraction quality, identifying transcripts of the gene of interest using reverse transcription PCR (RT-PCR) may be technically demanding. At the protein level, GOI specific antibodies or tagged antibodies can be utilized in Western blotting (WB) and indirect immunofluorescence assays (IFA) verify the expression of GOI. In addition, depending on the purpose of genetic modification, biological analyses of GM oocysts—such as morphology, oocyst shedding patterns, pathogenicity, and immunogenicity—can be systematically compared with those of the parental strain.

Genetic manipulation often aims to intentionally alter specific traits of an organism, a goal typically accomplished through the careful design of transfection constructs. Transfection involves the introduction of foreign DNA or RNA fragments into recipient cells under specific conditions, enabling the cells to adopt new phenotypes. Among the various vectors available, plasmids are the most common and reliable carriers of exogenous nucleic acid material.^30^ For the successful expression of foreign genes or the gene of interest (GOI) in eukaryotic recipient cells, essential components include a promoter and a complete open reading frame (ORF) (Figure 2). The promoter is particularly important as it regulates the initiation and level of gene transcription, as well as the spatial and temporal expression of the protein, making it a critical regulatory element in the design of transfection constructs. The promoters in eukaryotes generally do not have distinct sequence characteristics. In practice, the promoter is typically considered to be the sequence of about 1000 base pairs (bp) or longer upstream of the gene’s ORF. Promoter functions in regulating the expression of GOI are highly conserved among *Eimeria* species (Table 1). For example, the promoters of *E*. *tenella* histone protein 4 (EtHis4), surface antigen 13 (EtSAG13), and microneme protein 2 (EtMIC2) can successfully drive the expression of heterologous genes in other *Eimeria* species, such as *Eimeria mitis, Eimeria acervulina*, and *Eimeria necatrix* (Table 1). These promoters are also effective in species that infect mammals, including *Eimeria falciformis*, *Eimeria magna*, *Eimeria intestinalis*, and *Eimeria nieschulzi*, which infect mice, rabbits, and rats (Table 1). However, there are differences in how these promoters operate across various *Eimeria* species in different hosts. The EtSAG13 promoter fails to drive reporter gene expression in unsporulated oocysts of chicken-infecting species like *E*. *tenella* and *E*. *acervulina* but is active throughout all life stages of mammalian-infecting species such as *E. falciformis* and *E. magna*.^49^^,^^69^^,^^70^ This indicates that using the gene’s native promoter for genetic manipulation, especially in studies focused on development-related genes, is more effective in accurately uncovering the true expression patterns of the gene of interest. Moreover, promoter functions are conserved across other apicomplexan parasites. The promoters of *Toxoplasma gondii* tubulin (TgTublin) and surface antigen 1 (TgSAG1) can regulate gene expression in *E*. *tenella*. Conversely, the EtHis4 promoter can also regulate gene expression in *T*. *gondii*.^38^ This conservation of promoter functions offers more versatility in genetic manipulation across different *Eimeria* species, thereby aiding in the development of advanced genetic manipulation platforms for these parasites.

**Table 1:** Promoters for regulation of reporter gene expression in GM *Eimeria*

Promoter	*Eimeria* spp	Signal strength^a^	Transient Expression^b^	Stable Expression^c^	Reference
EtSAG1	*E. tenella*	+ + +	/	+	Yu et al.^31^
EtSAG13	*E. tenella*	+ + + + +	**−**	+	Tang et al.^32^; Tang et al.^33^; Tang et al.^34^; Tang et al.^35^
EtSAG13	*E. acervulina*	+ + + +	**−**	+	Liu et al.^36^
EtSAG13	*E*. *necatrix*	+ + + +	**−**	+	Duan et al.^22^; Duan et al.^37^
EtHis4	*E. tenella*	+ + + +	+	+	Liu et al.^30^; Zou et al.^38^; Clark et al.^39^; Hao et al.^40^; Hu et al.^41^; Liu et al.^42^; Shi et al.^43^; Shi et al.^44^; Su et al.^45^; Tang et al.^46^; Yan et al.^47^
EtHis4	*E*. *necatrix*	+ + + +	+	+	C.D., and X.T., unpublished data
EtHis4	*E. mitis*	+ + + +	+	+	Qin et al.^48^
EtHis4	*E*. *magna*	+ + + +	+	+	Tao et al.^49^
EtHis4	*E. intestinalis*	+ + + +	+	+	Shi et al.^50^
EtActin	*E. tenella*	+ + +	+	+	Zou et al.^38^; Shi et al.^44^; Tang et al.^46^; Yan et al.^47^; Clark et al.^51^; Huang et al.^52^; Marugan-Hernandez et al.^53^; Pastor-Fernandez et al.^54^; Yin et al.^55^
EtActin	*E*. *necatrix*	+ + +	+	+	C.D., and X.T., unpublished data
EtActin	*E. mitis*	+ + +	+	+	Qin et al.^48^; Li et al.^56^; Qin et al.^57^
EtActin	*E. nieschulzi*	+ + +	+	+	Chen et al.^58^; Hanig et al.^59^
EtMic1	*E. tenella*	+ + +	+	+	Clark et al.^39^; Clark et al.^51^; Huang et al.^52^; Pastor-Fernandez et al.^54^; Yin et al.,^55^Kelleher & Tomley^60^; Marugan-Hernandez et al.^61^; Pastor-Fernandez et al.^62^; Yin et al.^63^
EtMic1	*E. nieschulzi*	+ + +	+	+	^64^
EtMic2	*E. tenella*	+ + + +	+	+	Fernandez et al.^54^^,^Hernandez et al.^61^; Pastor-Fernandez et al.^62^; Kurth & Entzeroth^64^; Marugan-Hernandez et al.^65^
EtMic2	*E. acervulina*	+ + + +	+	+	Guo et al.^66^; Yu et al.^67^
EtMic2	*E. mitis*	+ + +	+	+	Qin et al.^57^
EtMic5	*E. tenella*	+ +	+	+	Hernandez et al.^61^
EtMic9	*E. tenella*	+ +	+	+	Hernandez et al.^61^
EtMCP2	*E. tenella*	+ + +	+	+	Marugan-Hernandez et al.^65^
EtTIF	*E. tenella*	+	+	+	Marugan-Hernandez et al.^53^; Hernandez et al.^61^
EaSAG1	*E. acervulina*	+ + +	/	+	Yu et al.^31^
EaMCP1	*E. acervulina*	+ + + +	/	+	Yu et al.^31^
EaMic2	*E. acervulina*	+ + + +	/	+	Yu et al.^31^
EaHAP2	*E. acervulina*	+	–	+	Liu et al.^36^
EtGAM56	*E. nieschulzi*	+ +	/	+	Chen et al.^58^; Hanig et al.^59^
EaGAM56	*E. acervulina*	+ +	–	+	Liu et al.^36^
EnOWP2	*E. nieschulzi*	+ +	/	+	Jonscher et al.^68^
EnOWP6	*E. nieschulzi*	+ +	/	+	Jonscher et al.^68^
TgTub	*E. tenella*	+ + +	+	+	Zou et al.^38^; Huang et al.^52^; Yin et al.^63^

a The signal strength indicates the author’s assessment of promoter activity based on fluorescent gene expression, where more “+” signs signify stronger promoter function.

b “/”: no *in vitro* transfection or inconclusive results; “−”: transfection conducted but no signal detected; “+”: signal or GOI expression detected.

c “+”: signal or GOI expression detected.

The role of the 3′ untranslated region (3′ UTR) in the expression of heterologous genes in recombinant *Eimeria* remains somewhat unclear, although it is likely linked to mRNA stability.^71^ Studies using *Plasmodium* as a model organism have shown that the absence of the 3′ UTR can reduce gene expression by over 90%.^71^ While the 3′ UTR is often given limited consideration in the design of transfection constructs for *Eimeria*, the commonly used 3′ UTR from the *E*. *tenella* actin (EtActin) gene has been found to function effectively across different promoters and genes.^31^^,^^46^^,^^49^^,^^63^^,^^67^

The expression of exogenous genes in *Eimeria* is tightly regulated by promoters, which also govern their stage-specific expression during various life cycle phases. The spatial distribution of these expressed genes within different subcellular compartments of recombinant *Eimeria* is influenced by both the characteristics of the heterologous protein and the regulatory sequences employed. For instance, the enhanced yellow fluorescent protein (EYFP), when driven by the EtSAG13 promoter, predominantly localizes to the refractile body (RB) of sporozoites during the sporulated oocyst stage.^33^^,^^34^ This specific localization may be due to the inherent properties of EYFP or the biological function associated with RB. When EYFP is fused with the nuclear localization sequence of EtHis4, it targets the nucleus of sporozoites.^42^^,^^55^^,^^72^ In contrast, when fused with the EtMIC2 protein or its signal peptide, EYFP is expressed in the micronemes of sporozoites.^31^ A fusion with the signal peptide from *T*. *gondii* dense granule protein 8 (TgGRA8) results in secretion into the parasitophorous vacuole.^31^^,^^55^^,^^63^ Furthermore, when fluorescent proteins are fused with a membrane protein’s anchoring sequence (GPI), the fluorescence signal is concentrated in the cell membrane of sporozoites.^31^^,^^57^ Thus, the choice of regulatory sequences is crucial when conducting functional studies on genes with different cellular localizations using genetic manipulation techniques.

### Recipient cells for transfection

*Eimeria* species exhibit a complex life cycle, with distinct cell types at various stages, including sporozoites, merozoites (where different species undergo varying numbers of schizogony cycles, each producing a different number of merozoites), male and female gametes, zygotes, unsporulated oocysts, and sporulated oocysts (each containing four sporocysts, with two sporozoites each). To date, several cell types have been used as recipient cells for transfection, including sporozoites, second-generation merozoites, unsporulated oocysts, and sporocysts (Figure 2). Among these, sporozoites are the most commonly utilized recipient cells for transfection, particularly in chicken *Eimeria* species such as *E*. *tenella,*^39^^,^^40^^,^^60^ *E*. *mitis*,^48^ and *E*. *acervulina*,^36^ as well as in rabbit and rodent *Eimeria* species such as *E. magna,*^49^ *E. intestinalis*,^50^^,^^70^ *E. falciformis*,^70^ and *E. nieschulzi*.^59^ Merozoites have only been reported as recipient cells in *E*. *necatrix*, providing a potential model for selecting transfection recipient cells in other *Eimeria* species.^22^ Transfection of oocysts has presented significant challenges in *Eimeria* genetic manipulation, although it is considered the most ideal recipient cell (as described in the life cycle section). Gene gun techniques have successfully introduced exogenous plasmids into the protoplasts of unsporulated oocysts of *E. maxima*, but the reporter gene failed to express.^15^ This failure is possibly due to the extensive damage inflicted by the gene gun, which inhibits sporogony, or due to challenges associated with promoter selection. Additionally, transfection using sporocysts as recipient cells has been successfully performed in rodent *Eimeria*, providing valuable insights for selecting recipient cells in the genetic manipulation of other *Eimeria* species.

### Reporters for transfection and selection

Reporter genes are introduced into recipient cells to produce detectable traits that are not naturally present in those cells.^73^ Typically, these genes have the following characteristics: (1) The gene sequence is well-documented; (2) the expression product does not exist in the recipient cell, ensuring no background interference; and (3) the expression product is easily measurable. The coding sequence of the reporter gene is fused with regulatory sequences or other target genes, allowing the detection of the reporter gene’s product to “report” the expression status of the target gene.

**Figure 3:**
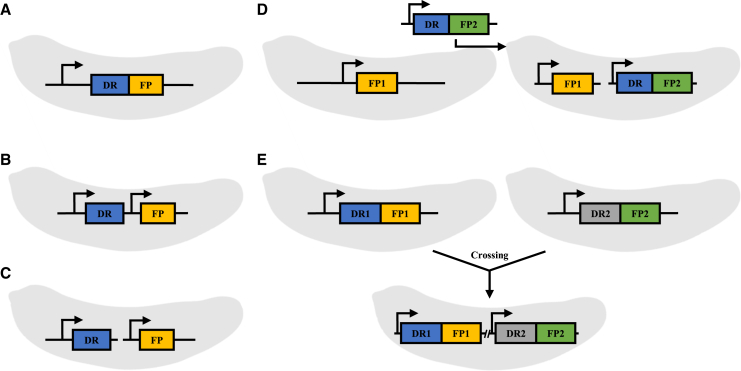
Schematic diagram of strategies to enhance the screening efficiency of GM *Eimeria* Due to the low transfection and screening efficiency in *Eimeria* species, it is common to combine multiple screening genes when selecting genetically modified parasites. Several strategies can be employed: (A) **Fusion expression of drug resistance gene (DR) and fluorescent reporter gene (FP):** These genes can be linked by a flexible peptide or a P2A sequence, enabling their simultaneous expression in one construct. (B) **Dual expression cassette plasmid:** This strategy involves placing the DR and FP in separate expression cassettes, allowing for the acquisition of both screening genes in a single transfection. (C) **Co-transfection of multiple plasmids:** Different screening genes, such as DR and FP, are constructed into separate plasmids and co-transfected, allowing for the expression of multiple screening genes in the resultant parasite. (D) **Serial genetic modification:** A stable strain expressing a single screening gene is used as the parental strain for further genetic modifications, introducing a new screening gene distinct from the original one. (E) **Genetic cross:** Parasite strains expressing different screening genes are crossbred. The progeny parasites expressing both parental screening genes are selected, achieving the co-expression of multiple screening genes. These strategies, tailored to specific experimental goals, can significantly enhance the efficiency of genetic manipulation in *Eimeria*. Currently validated drug resistance genes include mutated versions of *TgDHFR* and *EtPRS*, with the application of diclazuril-resistant mutants under investigation. Effective fluorescent reporter genes include EYFP, GFP, mCitrine, RFP, and mCherry.

Reporter genes can be categorized into three types based on the nature of the expression product and the detection methods used: (1) Direct detection: This category includes fluorescent protein reporter genes that emit a bright fluorescent signal under specific excitation wavelengths. Commonly used fluorescent protein genes in *Eimeria* include Green Fluorescent Protein (GFP), EYFP, mCitrine (a YFP variant), and Red Fluorescent Proteins (RFP and mCherry) (Figure 2). (2) Phenotypic changes: These reporter genes confer a specific phenotype to the recipient cell, the most common is drug resistance gene. Transfected cells that survive and proliferate under drug pressure are selected and enriched through continuous propagation. Recombinant *Eimeria* expressing the *T*. *gondii* dihydrofolate reductase mutant (TgDHFR-m2m3) gene can be selected using the drug pyrimethamine.^47^^,^^59^ Identifying mutation sites in genes that confer resistance to anticoccidial drugs like halofuginone, diclazuril (unpublished observation), and decoquinate provides additional options for reporter genes in *Eimeria* genetic manipulation.^5^^,^^26^^,^^74^^,^^75^ (3) Enzymatic reaction: These reporter genes produce a detectable signal when their expression product reacts with other components. For instance, β-galactosidase is a reporter gene in recombinant *Eimeria* that catalyzes the hydrolysis of β-galactosides, reacting with 5-bromo-4-chloro-3-indolyl β-D-galactopyranoside (X-gal) to produce a yellow product, *o*-nitrophenol, which can be qualitatively or quantitatively measured. This was the first reporter gene used to demonstrate the feasibility of genetic manipulation in *Eimeria*.^60^ However, the technical challenges of culturing *Eimeria in vitro* have limited the use of β-galactosidase as a reporter gene, leading to a focus on fluorescent proteins and drug resistance genes as the primary reporter genes. Occasionally, these two types of reporter genes are combined to enhance the screening efficiency of recombinant *Eimeria* (Figure 3).

### Transfection system

The efficiency of genetic manipulation in *Eimeria* is influenced by several critical factors, including the choice of genetic material carriers, the type of recipient cells, buffer solutions, and the transfection method used. Currently, the most commonly employed transfection method for *Eimeria* is physical transfection technique, specifically electroporation. When electroporating *Eimeria* sporozoites, both plasmids and PCR fragments can serve as carriers for the genetic material.^39^ Typically, about 25 μg of DNA is used, corresponding to approximately 5×10^6^ sporozoites. Assuming a DNA fragment size of 5000 bp, each sporozoite would receive about 10^6^ copies of the DNA fragment. The transfection buffer used is CytoMix, with a volume of 800 μL, and the electroporation conditions are set to 1.5 kV, 25 μF, using square wave parameters (for example, with a Bio-Rad Gene Pulser, although specific settings may vary depending on the instrument or laboratory conditions). Under these conditions, the transfection efficiency is generally low, around 0.00001%. However, adding restriction endonucleases to the transfection system can enhance transfection efficiency by approximately 200-fold.^30^ This improvement has also been observed in *Toxoplasma* and *Plasmodium*, though the exact mechanism remains unclear.^76^^,^^77^ It is speculated that restriction endonucleases may create nicks in the recipient cell genome, which facilitates the integration of exogenous DNA fragments. Lonza’s patented Nucleofector nuclear transfection technology, which integrates optimized electroporation instruments, buffers, and programs, can deliver foreign nucleic acids into the cytoplasm and directly into the nucleus, significantly improving transfection efficiency. The optimal transfection program for *Eimeria* using this system is U-033.^39^ The Nucleofector system and equipment are continuously evolving, with the 4D system being one such advancement, which is expected to further enhance transfection efficiency in *Eimeria*.

### Observation and selection of genetically modified *Eimeria*

*Eimeria* sporozoites can invade primary chicken kidney cells (PCKC), Madin-Darby bovine kidney (MDBK) cells, and Vero cells from African green monkeys, where they undergo development.^33^^,^^64^^,^^78^ Transfected sporozoites inoculated into these cells allow for easy observation of reporter genes (Figure 2), making this a commonly used transient transfection system for screening promoters, reporter genes, and optimizing transfection conditions. This system also serves as the first step in preliminarily determining the success of genetic manipulation. However, due to the difficulty of completing the life cycle of *Eimeria in vitro*, obtaining a large number of genetically modified (GM) recombinant parasites for further study requires the acquisition of oocyst-stage parasites for continuous passage in animals. This necessitates the stable integration of exogenous genes into the *Eimeria* genome, a process known as stable transfection.

Different inoculation methods are used to obtain GM *Eimeria* oocysts based on the type of recipient cells and the site of infection. For instance, sporozoites of *E*. *tenella* and *E*. *mitis*,^40^^,^^48^^,^^60^ which parasitize the cecum, can be successfully inoculated through the cloaca (Figure 2). For *E*. *necatrix*, where schizogony involves some migration, transfected merozoites can also be inoculated through the cloaca to obtain GM oocysts.^22^ It is important to note that due to the high virulence of *E*. *tenella* and *E*. *necatrix*, the inoculation dose should be strictly controlled during transfection to avoid the death of the inoculated chickens, which would prevent the recovery of GM oocysts. *E*. *acervulina*, which parasitizes the duodenum, cannot be inoculated through the cloaca; instead, transfected sporozoites can be inoculated via the wing vein.^79^ Though this method, especially in chicks, requires a certain level of technical proficiency to ensure successful inoculation (Figure 2). For rabbit-infecting species like *E*. *magna* and *E*. *intestinalis*, transfected sporozoites are surgically inoculated into the duodenum to obtain GM oocysts.^49^^,^^50^ Some researchers have also used an oral inoculation method, where animals are first given a small amount of alkaline solution to neutralize stomach acid before orally inoculating the sporozoites. Although no systematic comparison of different inoculation methods has been conducted, laboratory experience suggests that this method is less stable than cloacal, venous, or surgical inoculation. However, if transfection could be successfully achieved in oocysts and GM oocysts obtained through oral inoculation, it would represent a milestone in the field of *Eimeria* genetic manipulation.^80^

The transfection of *E*. *tenella* using a β-galactosidase reporter gene was first reported in 1998.^60^ Due to the lack of a screening system based on this reporter gene, progress in *Eimeria* genetic manipulation stalled for nearly a decade. The application of fluorescent protein reporter genes in *Eimeria* genetic manipulation, along with flow cytometry sorting techniques based on fluorescent proteins,^47^^,^^55^ has enabled effective selection of GM parasites (Figure 2). Stable expression of fluorescent proteins in GM parasites can be achieved after 5–7 generations of continuous propagation.^47^^,^^55^ The combination of drug selection and flow sorting significantly improves the efficiency of selecting GM parasites,^56^^,^^59^ reducing the selection time by more than half (Figure 2). The combination of two selection strategies can be achieved through approaches such as dual-expression cassette transfection constructs, co-transfection, or genetic crossing (Figure 3). The specific strategy should be flexibly chosen or combined based on the goals of genetic manipulation.

With the mutation sites of resistance genes to commonly used anticoccidial drugs are identified, more combinations and optimization schemes for selecting recombinant parasites become available.^5^^,^^74^^,^^75^ Previous research showed that the number of sporocysts expressing reporter genes within a GM sporulated oocyst could be 1, 2, 3, or 4, meaning not all oocysts expressing the reporter gene would release 100% positive sporocysts.^39^ This reduces the efficiency of obtaining GM parasites through oocyst-based flow sorting. Recent studies have shown that sporocyst-based flow sorting is significantly more effective in enhancing the selection efficiency of GM parasites compared to oocyst-based sorting.

### Gene editing systems and extensions

The CRISPR/Cas9 system is a powerful gene-editing tool widely used across various species, including some Apicomplexan parasites such as *Toxoplasma, Plasmodium, Cryptosporidium*, and *Leishmania*.^81^^,^^82^^,^^83^^,^^84^ The CRISPR/Cas9 system could also be applied to *Eimeria* gene editing through the following four aspects: (1) The EtHis4 promoter and nuclear localization sequence can regulate the expression of the *Streptococcus pyogenes* cas9 (SpCas9) nuclease in the sporozoite nucleus, providing the basis for SpCas9 to cleave the nuclear genome of the parasite; (2) *E*. *tenella* has two U6 promoters, both of which can effectively regulate the expression of sgRNA, and the expressed sgRNA shows biological activity; (3)This system mediates the formation of double-strand breaks (DSBs) at the target site in the *Eimeria* genome; (4) The DSBs in the *Eimeria* genome can undergo homologous recombination repair in the presence of homologous fragments.^72^ Subsequent researchers have explored two main ways to improve gene editing efficiency: one is constructing GM *Eimeria* strains that stably express SpCas9, using these as parental strains for gene editing to reduce the impact of low efficiency in co-transfection with multiple plasmids.^29^ Another one is RNP-mediated gene editing, where *Francisella novicida* FnCas12a protein is incubated with guide RNA *in vitro* to form a ribonucleoprotein (RNP) complex, which is then transfected to achieve gene editing.^85^ Each of these gene-editing systems has been successfully applied, with different methods offering distinct advantages and disadvantages, suitable for various applications.

With the application of the CRISPR/Cas9 system, further extended applications have also been reported. For example, the CRISPR/Cas system has been used in molecular diagnostics, primarily based on its ability to specifically recognize and cleave nucleic acid sequences. The *trans*-cleavage activity of Cas proteins (Cas12, Cas13, and Cas14) can amplify detection signals and improve detection sensitivity and specificity.^86^^,^^87^^,^^88^ This has led to the establishment of identification and diagnostic techniques for different *Eimeria* species. Additionally, the CRISPR/Cas9 system has been applied to epigenetic research in *Plasmodium* and *Toxoplasma* and adapting this system for *Eimeria* epigenetic research holds significant value.^89^^,^^90^

### Evaluation system

The stability of GM *Eimeria* is fundamental for studying its biological, genetic, and immunological properties. This necessitates a systematic evaluation of the stable expression of heterologous genes within recombinant *Eimeria*. Researchers have established various systems for identifying the expression of heterologous genes at the genomic, transcriptional, and protein expression levels. For example, genome walking and Southern blot techniques are used to identify the insertion sites and copy numbers of heterologous gene fragments in the recombinant *Eimeria* genome.^33^^,^^42^^,^^45^ With the advent of high-throughput sequencing technologies, analyzing the insertion sites of heterologous genes using next-generation sequencing (NGS) is also a valuable research direction. If site-specific integration is achieved using gene-editing technologies such as CRISPR/Cas9, PCR amplification and sequencing can accurately analyze the genomic insertion sites and targeting efficiency.^41^^,^^72^ Assessing the expression of foreign genes at the transcriptional level is challenging because genetic manipulation typically involves only the coding regions of foreign genes, excluding introns, which require careful RNA extraction to minimize DNA contamination during reverse transcription. Compared to transcriptional analysis, evaluating protein expression levels in recombinant *Eimeria* is more straightforward. Fluorescent proteins can be used as reporter genes fused with foreign genes, allowing the expression of foreign genes to be monitored through fluorescent signal detection.^22^^,^^31^^,^^67^ Additionally, commonly used protein tags like Flag, HA, and Ty, along with their corresponding commercial antibodies, can be applied to identify heterologous gene expressions in GM *Eimeria*.^35^^,^^41^ In cases where antibody specificity is suboptimal, mass spectrometry can effectively detect heterologous proteins expressed in recombinant *Eimeria*.^67^

## Technical bottlenecks in the genetic manipulation of Eimeria

The platform for genetic manipulation of *Eimeria* has seen significant progress over the past 30 years due to the sustained efforts of researchers worldwide. However, several challenges remain, hindering the basic biological and applied research of *Eimeria*. These include: (1) Lack of efficient *in vitro* culture systems: The absence of a robust *in vitro* culture system increases the difficulty and cost of genetic manipulation experiments. This limitation is especially significant in the study of the *Eimeria* species that infect large animals such as pigs, cattle, and sheep. Some progress has been made with the establishment of an *in vitro* culture system for *Isospora suis* (a coccidian parasite in pigs) using porcine intestinal epithelial cells, which allowed the development of sporozoites to mature schizonts.^91^ This system showed potential by enabling the sexual development phase of *Isospora* without host cells, culminating in oocyst formation. These advancements offer valuable insights for similar research on *Eimeria*. (2) Low transfection efficiency: Compared to other apicomplexan parasites such as *T*. *gondii, Eimeria* has a relatively low transfection efficiency. There is a need for systematic optimization of the transfection process, including the transfection system and ratios, buffers, and the selection of recipient cells. (3) Low screening efficiency for clonal populations of recombinant parasites: The current understanding is that clonal populations derived from a single sporocyst are considered clones. However, the immune response of the host and uncertainties during the inoculation process make it challenging to obtain a monoclonal population.^33^^,^^42^^,^^47^ (4) Lack of adequate screening genes: Some drugs are suitable for *in vitro* culture systems, but conducting animal experiments is costly. Although the resistance mutation sites for commonly used anticoccidial drugs have been gradually identified, verification of these findings is time-consuming.^75^^,^^92^^,^^93^ These technical bottlenecks limit the application of highly efficient genetic manipulation techniques in *Eimeria* research, highlighting the urgent need for breakthroughs in this field.^94^

## The application of genetic manipulation techniques in *Eimeria*

### Study of parasite gene’s function using transgenic parasites

The development of transgenic parasites, based on genetic manipulation systems, is the basis for studying the function of *Eimeria* genes. First, recombinant *Eimeria*, expressing a fluorescent protein reporter gene, was used to observe the life cycle of *Eimeria* and can clearly reveal the morphology of *Eimeria* at different developmental stages.^43^^,^^48^^,^^55^ The recombinant *Eimeria* expressing fluorescent protein reporter genes can clearly observe the results that are difficult to be detected by tissue sections and intestinal smear, which has significant advantages in observing the changes in protoplasm and the formation of sporocysts during *in vitro* sporogony of *Eimeria*.^58^^,^^68^

Second, the establishment of *Eimeria* gene editing technology and the application of transgenic parasites make it possible to study the biological mechanism of *Eimeria* and analysis gene function at the molecular level.^41^ The AP2 transcription factor was found to play an important role in the growth and development of *Eimeria* by genetic manipulation of sporulation stage specific transcription factors.^27^^,^^41^

Third, genetic manipulation techniques for mining and validating the application of drug resistance gene mutations provide an effective method.^5^^,^^6^^,^^26^^,^^75^ By using genetic manipulation technology to introduce resistance mutation site genes into sensitive strains, researchers can obtain drug resistance. Conversely, gene editing technology can be applied to restore the resistance mutation site in resistant strains, thereby reverting them to drug sensitivity. This approach has been used to analyze the resistance mutation sites of halofuginone and other drugs, providing a solid foundation and exemplar for verifying other anticoccidial drugs resistance sites.^5^^,^^75^

### Application of recombinant *Eimeria* as a vaccine vector

Transgenic *Eimeria* serves as an optimal coccidia antigen delivery vehicle and signifies a novel class of vaccines.^18^ It is a crucial research focus to design and refine recombinant *Eimeria* as a vaccine vector, with the goal of improving the protective effect of vector vaccines. Recombinant *Eimeria* has been investigated as a vaccine vector for expressing antigens from parasites, bacteria, and viruses, showing a broad and promising potential for application.^18^

#### Expression of parasite protective antigens

Researchers have constructed a transgenic *E*. *tenella* (Et-TgSAG1) expressing the surface antigen 1 of *T*. *gondii* (TgSAG1) and have confirmed that it can elicit both TgSAG1-specific immune responses in chickens and mice. Also, in the mouse model, vaccination with Et-TgSAG1 extended the survival period post-infection. It suggests that Et-TgSAG1 has the potential to serve as a recombinant vaccine vector against toxoplasmosis in avian and mammalian species.^46^ A novel vaccine delivery system using transgenic *E*. *tenella* expressing protein 1 of *E*. *maxima* (Et-EmIMP1). Et-EmIMP1 can stimulate chicks to produce specific cellular and humoral immunity responses, thereby partially protecting chicks from infection by the sensitized *E*. *maxima* and *E*. *tenella*.^34^

#### Expression of bacterial protective antigens

*E*. *tenella* has been treated as a novel vaccine vector, expressing the exogenous antigen CjaA to achieve immune protection against *Campylobacter jejuni* in the chicken intestines. The authors have successfully constructed transgenic *Eimeria* that express both CjaA and fluorescent protein and have confirmed its sustained expression of the CjaA gene. A single or multiple using of these transgenic *Eimeria* significantly reduced the colonization of *C. jejuni* in chickens, achieving high protection, with a significant difference compared to both unvaccinated groups and *E*. *tenella*.^51^

#### Expression of viral protective antigens

Researchers successfully constructed the recombinant *Eimeria* expressing IBDV VP2 and ILTV gI by using the gene-edited *E*. *tenella* to express the viral antigens of IBDV and ILTV, and specific antibody responses were detected by western blot and ELISA.^53^ The recombinant *Eimeria*, generated by fusing IBDV VP2 and *Eimeria* microneme, stimulated a specific antibody response in the host, resulting in a significantly reduced impact of IBDV upon challenge infection.^67^ When an increase in a copy of the VP2 gene from IBDV, immune responses are enhanced.^66^

## Concluding remarks and future perspective

In today’s rapidly growing poultry industry, coccidiosis remains one of the key factors limiting animal health and farming efficiency, making it a priority disease for control. While the large-scale, regular use of anticoccidial drugs has suppressed the development and spread of coccidiosis to some extent, it is insufficient to eliminate or eradicate the disease. Vaccines based on live oocyst components have been in clinical use for over 70 years; however, side effects of live vaccines persist. Genetic manipulation of existing coccidiosis vaccine strains represents a promising direction for the development of novel vaccines. (1) Development of recombinant *Eimeria* expressing heterologous protective antigens: By constructing recombinant *Eimeria* strains that express protective antigens from heterologous species, vaccines with cross-immunity can be developed. This approach reduces the number of components in live coccidiosis vaccines, especially the inclusion of highly pathogenic strains, thereby enhancing vaccine safety.^32^^,^^34^^,^^54^ However, breakthroughs are needed in the identification of *Eimeria* protective antigens and their expression in recombinant strains. (2) Construction of recombinant *Eimeria* expressing molecular adjuvants: Enhancing the immunogenicity of certain strains through molecular adjuvants can reduce immunization dosages and improve vaccine safety. For example, the expression of secretory IL-2 or profilin and the display of antibody Fc fragments on the sporozoite membrane surface both enhanced the immunogenicity of recombinant *Eimeria* to some extent.^35^^,^^56^^,^^57^ Other types of molecular adjuvants may also enhance the immunogenicity of *Eimeria* antigens, and a rational combination with existing molecular adjuvants holds the potential for a significant increase in immunogenicity, this requires precise temporal and spatial design of adjuvant expression. (3) Targeting core genes regulating sporogony: By identifying and manipulating core genes involved in sporogony development, vaccine strains with defective sporogony can be constructed. Following vaccination, the sporogony of oocysts excreted by the next generation is significantly reduced, minimizing the risk of chickens ingesting high doses of vaccine-derived sporulated oocysts and thereby reducing the side effects of vaccination.^27^

The development of genetic manipulation techniques will facilitate the transition of *Eimeria* species from non-model to model organisms for apicomplexan parasites research. The Apicomplexa phylum includes numerous protozoan parasites with complex life cycles. *Eimeria*, which completes its entire life cycle in a single host, serves as an ideal model for studying developmental regulation, particularly mechanisms of sexual reproduction. Constructing strains that express fluorescent proteins specific to the sexual stage, combined with flow cytometry and omics-based selection technologies, can help identify a set of regulatory genes for sexual reproduction. This will not only elucidate the biological functions and mechanisms of these genes but also provide a reference for the study of sexual reproduction mechanisms in other Apicomplexa parasites, such as *T*. *gondii*. Furthermore, by applying continuous selective pressure for early oocyst shedding, genetically stable precocious lines of *Eimeria* with significantly shortened prepatent periods can be obtained. This is a key technical principle for developing attenuated live vaccines.^95^ The stability of precocity, the ease of obtaining genetic material, and the short reproductive cycle make *Eimeria* a promising model organism for genetic studies on lower organisms and developmental regulation.

With the establishment and continuous optimization of genetic manipulation platforms, particularly CRISPR/Cas9-based gene-editing systems, research into the biological and immunological characteristics of *Eimeria* is poised to achieve new breakthroughs. Future key research directions for genetic manipulation of *Eimeria* include but are not limited to the following: (1) Development of conditional gene knockout systems. Conditional knockout systems are essential tools for studying indispensable genes. Examples include genome-level conditional knockout systems like the Cre/DiCre-loxP system and the tetracycline-inducible system, transcription-level systems such as the U1 small nuclear ribonucleic particles-mediated gene silencing system, and protein-level systems such as the auxin-based degron system, the destabilization domain (ddFKBP) system, and light-inducible systems. These systems have been successfully or partially applied in *T. gondii*, *Plasmodium* spp., and *Cryptosporidium* spp.^96^^,^^97^^,^^98^^,^^99^^,^^100^ However, their application in *Eimeria* has not yet been reported, primarily due to the high cost of certain drugs or reagents required for use in host animals. Finding alternative, cost-effective drugs or reagents and accelerating the establishment of *Eimeria in vitro* culture systems will be critical for advancing this field. (2) Construction and application of artificial chromosome systems. Artificial chromosome systems in bacteria and fungi have demonstrated significant advantages in the stable transfection of large DNA fragments and genetic manipulation.^101^^,^^102^ By analyzing sequences such as centromeres and telomeres, artificial chromosome systems have also been successfully established in *Plasmodium* spp.^103^ With the completion of *Eimeria* genome data, developing an artificial chromosome system for *Eimeria* could greatly advance the study of its biological and genetic characteristics. Moreover, it would facilitate the development of novel vaccine strains for coccidiosis, the use of *Eimeria* as a vaccine vector improving the efficient expression of exogenous pathogenic genes. The continuous improvement of genetic manipulation techniques will provide a solid technical foundation for the development of novel formulations for coccidiosis control.^104^ Additionally, it will facilitate the advancement of *Eimeria* as a new model organism for studying important biological and genetic mechanisms, such as sexual reproduction and precocious development.

## Acknowledgments

This study was supported by Beijing Natural Science Foundation (6232032), 10.13039/501100001809National Natural Science Foundation of China (32273035 and 31902295) and the Youth Innovation Program of Chinese Academy of Agricultural Sciences (Y2023QC10).

## Author contributions

**Y.L.:** Writing – original draft. **J.S.:** Writing – review and editing. **R.L.:** Writing – original draft, writing – review and editing. **L.L.:** Writing – review and editing. **X.L.:** Writing – review and editing. **J.D.:** Writing – review and editing. **X.S.:** Conceptualization, writing – review and editing. **X.T.:** Conceptualization, writing – original draft, writing – review and editing.

## Declaration of interests

The authors declare no declaration of interest.
